# Body mass index affects the association between plasma lipids and peripheral eosinophils in a general chinese population: a cross-sectional survey

**DOI:** 10.1186/s12944-023-01909-w

**Published:** 2023-09-07

**Authors:** Yu Gao, Xiaocheng Wang, Lan Gao, Xin Li, Weihong Bai

**Affiliations:** 1 Department of Otorhinolaryngology, Shanxi Provincial People’s Hospital Affiliated to Shanxi Medical University, No. 29 of Twin Towers Temple Street, Taiyuan, Shanxi 030012 China; 2 Department of Statistics, Shanxi Provincial People’s Hospital Affiliated to Shanxi Medical University, No. 29 of Twin Towers Temple Street, Taiyuan, Shanxi 030012 China; 3 College of Basic Medicine, Shanxi University of Chinese Medicine, Jinzhong, Shanxi 030619 China

**Keywords:** Plasma lipid, Body mass index, Peripheral blood eosinophil

## Abstract

**Background:**

Lipid metabolism affects type 2 immunity; however, the association between plasma lipids and eosinophilic inflammation in humans is uncertain. This study analysed the relationship between plasma lipids and peripheral eosinophils and whether patterns differ with different body mass indexes (BMI).

**Methods:**

A cross-sectional survey including 62,441 healthy participants recruited from a regular health screening programme was conducted. Participants were divided into normal weight, overweight and obese subgroups according to BMI.

**Results:**

Multiple linear regression analysis revealed that elevated logarithmic-transformed eosinophil counts (log(EOS)) significantly correlated with high total cholesterol(TC), triglyceride(TG), low-density lipoprotein-cholesterol (LDL-C), and low high-density lipoprotein-cholesterol (HDL-C)levels in the overall population, as well as in men and women, while certain associations between peripheral blood eosinophil percentage and serum lipids varied by gender. These correlations existed across almost all BMI subgroups, and standardised β values decreased sequentially with increasing BMI. HDL-C had the most significant effect on eosinophils in obese women. Two-factor analysis of variance showed log(EOS) increased with higher BMI and hyperlipidemia whether in male or female and a synergistic effect exists of lipid levels (TG and LDL-C) and BMI in men.

**Conclusions:**

Blood eosinophil counts were correlated with blood lipid levels and modified by body mass index status. The effects of lipid levels and body mass index on blood eosinophil counts were synergistic. Therefore, lipid metabolism may be involved in systemic eosinophil inflammation.

**Supplementary Information:**

The online version contains supplementary material available at 10.1186/s12944-023-01909-w.

## Background

Eosinophils are important players in type 2 immune responses such as allergic rhinitis, asthma, and eosinophilic chronic sinusitis. An elevated eosinophil count in the peripheral blood can indicate the eosinophil phenotype of chronic sinusitis [1] and is a risk factor for chronic obstructive pulmonary disease and decreased lung function [2]. Over the past 20 years, the incidence rates of eosinophil-related diseases have increased in East Asia. For instance, the proportion of eosinophilic nasal polyps in adults in central China increased from 15.7% in 2000 to 44% in 2015 [3]. However, the mechanism of this evolution remains unclear, thereby affecting the overall control of these diseases.

With the increasing incidence of obesity and metabolic syndromes, the relevance of metabolism to inflammation and immunity has received attention. Obesity and metabolic diseases, especially hyperlipidaemia, can trigger innate and adaptive immune responses [4, 5], which are evident in respiratory inflammation and allergic diseases. High low-density lipoprotein-cholesterol (LDL-C) and triglyceride (TG) levels are associated with high rates of asthma and aeroallergen sensitisation [6]. Low high-density lipoprotein-cholesterol (HDL-C) levels clinically predict high peripheral eosinophil count in Chinese patients with chronic obstructive pulmonary disease [7]. Patients with allergic rhinitis also present with dyslipidaemia, which aggravate the symptoms of allergic rhinitis [8] and increase the risk of chronic rhinosinusitis [9]. More importantly, patients with allergic rhinitis with different immunotherapy outcomes exhibit different fatty acid metabolic profiles [10], and dietary fatty acid intake may influence the development of immune tolerance [11]. These evidence suggest a close relationship between lipid metabolism and a type 2 immune response, especially eosinophilic inflammation; however, the mechanisms underlying this relationship remain largely unknown.

To elucidate the association between eosinophilic inflammation and lipid metabolism and assess the risk factors for eosinophil-associated diseases, providing evidence for the correlation between peripheral blood eosinophils and lipids in the general population is critical. However, most of the current literature concerning metabolism and immunity are derived from studies on cardiovascular diseases, focusing on the relationship between neutrophils or monocytes and lipids, with fewer studies examining eosinophils. Further conflicting results have been reported. A prospective lifeline cohort study in the Netherlands [2] and follow-up programme in a Japanese community-based population [12] reported that high eosinophil counts were related to low HDL-C levels and high LDL-C, TG, and total cholesterol (TC) levels. In contrast, data from a US multi-ethnic cohort showed no significant association between the level of each lipid and eosinophil count [13], and results from a white British cohort showed a negative association of TC and LDL-C with eosinophil count [14].

Therefore, this large-scale cross-sectional survey was conducted to determine the relationship between blood eosinophil counts and lipid profile in a general population in northern China. Considering that obesity and dyslipidaemia often coexist, and a non-linear positive correlation exists between body mass index (BMI) and eosinophil count [15], this study further analysed changes in this relationship according to BMI. Our overall aim was to better explore the pathogenesis of eosinophil-related diseases from a metabolic perspective and discover novel strategies to treat or prevent such diseases.

## Methods

### Study participants

Shanxi Provincial People’s Hospital has 2,300 beds, and its Health Screening Centre undertakes health examinations for the entire province. The centre cooperates with communities and work units to conduct comprehensive assessments concerning the health status of the general population. All resident Han Chinese individuals(The Han are the dominant ethnic group in China and share similar dietary habit) aged ≥ 18 years who underwent a health examination between January and December 2021 were invited to participate in this survey. In total, 69,494 participants were included. Among them, 2,068 participants (2.98%) were excluded because of missing relevant data; 3,293 (4.74%) due to known familial hyperlipidaemia, acute infections, injuries, haematological disorders, parasitic diseases, chronic pulmonary/liver/kidney diseases, malignant tumours, or abnormal or atypical leukocytes; 94 (0.14%) due to oral medications that affect the haematological system (31 patients received adrenal glucocorticoids, 10 estrogen/progesterone, 7 cyclosporin A, 14 JAK inhibitors, 11 retinoic acid, 9 methotrexate, 7 azathioprine and 5 tacrolimus); 136 (0.20%)with alcoholism; and 166 (0.24%) who were pregnant. In addition, 1296 individuals with BMI < 18.5 kg/m^2^ were excluded due to the tendency to increase dilution bias for the large sample size gap compared with other subgroups. Finally, 62,441 individuals were included in the current cohort. Acute infections were referred as self-reported fever, upper and lower respiratory tract infections, allergy/asthma attacks, dental/dermal infections, urinary tract infections, and diarrhoea within the past 2 weeks. This study was approved by the Ethics Committee of Shanxi Provincial People’s Hospital, China (2022148), and strictly abided by the tenets of the Declaration of Helsinki, including the guidelines for confidentiality and anonymity. Signed written consent was obtained from all participants.

### Measurement

Information regarding demographics, socioeconomic status, smoking/medical history, and current medications were collected via questionnaire. Blood pressure in the right arm was measured in a resting sitting position. After an overnight fast, venous blood samples were collected for complete blood count analysis and biochemical blood tests, including fasting blood glucose (FBG), TC, HDL-C, LDL-C, TG, glutamate-pyruvate transaminase, glutamate-oxaloacetate transaminase, urea nitrogen, and serum creatinine. BMI was calculated as weight/height squared (kg/m^2^). Participants were divided into four subgroups according to BMI standards in China: underweight, < 18.5; normal weight, 18.5–23.9; overweight, 24–27.9; and obese, ≥ 28 kg/m^2^. The diagnostic criteria for hyperlipidaemia were as follows: high TG level, ≥ 1.7 mmol/L(150 mg/dl); high TC level, ≥ 5.72 mmol/L(220 mg/dl); high LDL-C level, ≥ 3.4 mmol/L(130 mg/dl); and low HDL-C level, < 1.0 mmol/L(40 mg/dl).

### Statistical methods

SPSS 23 (IBM, Armonk, NY) was used for statistical analyses. Count data are presented as case numbers or percentages, and measurement data following a normal distribution are described as x̅ ± *s*; those not following a normal distribution are described as medians (interquartile range). Logarithmic (log) transformation was performed before analysis for variables with skewed distributions to improve data normality. The eosinophil count was skewed and conformed to normality after log transformation. The relationship between blood lipid levels and log-transformed eosinophil count (log[EOS]) or eosinophil percentage was evaluated using multiple linear regression in the overall population and each BMI subgroup. Two-factor analysis of variance (ANOVA) was used to assess the differences in log(EOS) at different blood lipid levels and BMI levels and to analyse the interaction between blood lipid levels and BMI. Data were adjusted for potential confounding effects, including sex; age; smoking status; BMI; WBC count; diastolic blood pressure; systolic blood pressure; levels of FBG, glutamate-pyruvate transaminase, glutamate-oxaloacetate transaminase, urea nitrogen, and serum creatinine; and the use of lipid-lowering medications. A further gender -stratified analysis was performed and menopausal status was treated as an adjustment variable given the significant effect of menopause on the lipid profile shift in women. A P-value of < 0.05 was considered statistically significant.

## Results

The basic characteristics of the study participants are presented in Table 1. This study comprised 62,441 participants aged 18 to 82 years. The median eosinophil count was 0.10 × 10^9^ cells/L. The median and interquartile range values were within the normal reference ranges of blood leukocytes for adults (0.05–0.5 × 10^9^ cells/L). The mean BMI value of the total population was 25.04 ± 3.32 kg/m^2^ and were within the overweight range. All lipid parameters were within the normal range. The prevalence of high TG, high LDL-C, high TC, and low HDL-C levels was 35.05%, 24.00%, 13.72%, and 7.07%, respectively. The prevalence of hyperlipidemia is 45.4% in men, 39.8% in women and 58.6% in postmenopausal woman. Among all participants, only 3431 (5.49%) reported the regular use of lipid-lowering medications. 2004(3.21%) take statin, 1105(1.77%) take fibrates and 87(0.14%) were treated with statatin and fibrate. The rest use Chinese medicine.

**Table 1:** Clinical parameters of the study participants (n = 62,441)

Variables	Mean ± SD, median (interquartile range), or n (%)
Sex	
Male	35,275 (56.5%)
Female	27,166 (43.5%)
Age (years)	47.42 ± 14.83
Body mass index (kg/m^2^)	25.04 ± 3.32
Normal	24,881 (39.8%)
Overweight	26,430 (42.4%)
Obese	11,130 (17.8%)
Smoking	
never moking	39,370 (63.1%)
current smoking	8798 (14.1%)
Heavy smokers(≥20 cigarettes/day)	2329 (3.7%)
former smoking	14,273 (22.9%)
Systolic pressure (mmHg)	123.94 ± 17.32
Diastolic blood pressure (mmHg)	75.02 ± 11.62
Fasting blood glucose (mmol/L)	5.58 ± 1.35
Glutamate-pyruvate transaminase (IU/L)	19.48 (13.83–29.25)
Log glutamate-pyruvate transaminase	1.32 ± 0.25
Glutamate-oxaloacetate transaminase (IU/L)	19.97 (16.89–24.28)
Log glutamate-oxaloacetate transaminase	1.32 ± 0.14
Urea nitrogen (mmol/L)	4.79 ± 1.27
Serum creatinine (µmol/L)	71.86 ± 18.59
Total cholesterol (mmol/L)	4.71 ± 0.95
Triglycerides (mmol/L)	1.68 ± 1.22
High-density lipoprotein-cholesterol (mmol/L)	1.25 ± 0.28
Low-density lipoprotein-cholesterol (mmol/L)	2.94 ± 0.70
Peripheral blood Eosinophils	
Percentage (%)	2.02 ± 1.46
Count (× 10^9^/L)	0.10 (0.06–0.16)
Log eosinophil count	−1.12 ± 0.57

SD, standard deviation

Tables 2 and 3 show the results of the multiple linear regression analysis of log(EOS) and eosinophil percentage, respectively, regarding lipid parameters in the overall population. Elevated log(EOS) values were significantly correlated with high TG, TC, and LDL-C levels and low HDL-C levels after adjusting for potential confounders (Table 2). Log(EOS) increased by 0.022, 0.018, and 0.045 for each unit increase in TC, TG and LDL-C levels, respectively. In contrast, it decreased by 0.039 for each unit increase in HDL cholesterol level (all p < 0.001). Additionally, after adjusting for potential confounders, peripheral blood eosinophil percentage positively correlated with TG, TC, and LDL-C levels, independent of the HDL-C levels (Table 3). The relevance of TC and LDL-C levels to EOS were relatively evident, with β coefficients of 0.043 and 0.054, respectively.

**Table 2:** Multiple linear regression results of log-transformed eosinophil count with lipid parameters in the overall population

Independent variable	β	SE	STDβ	P	95% CI
Total cholesterol	0.022	0.002	0.037	< 0.001	0.018 ~ 0.027
Triglycerides	0.018	0.002	0.037	< 0.001	0.014 ~ 0.021
High-density lipoprotein-cholesterol	−0.039	0.009	−0.019	< 0.001	-0.057~ -0.022
Low-density lipoprotein-cholesterol	0.045	0.003	0.056	< 0.001	0.039 ~ 0.052

The adjusted variables included sex, age, body mass index, WBC count, smoking, diastolic blood pressure, systolic blood pressure, fasting blood glucose, log glutamate-pyruvate transaminase, log glutamate-oxaloacetate transaminase, urea nitrogen, serum creatinine, and use of lipid-lowering medications

SE, standard error; STD, standardised; CI, confidence interval

**Table 3:** Multiple linear regression results of eosinophil percentage with lipids parameters in the overall population

Independent variable	β	SE	STDβ	P	95% CI
Total cholesterol	0.043	0.006	0.028	< 0.001	0.030 ~ 0.055
Triglycerides	0.012	0.005	0.010	0.027	0.001 ~ 0.022
High-density lipoprotein-cholesterol	0.029	0.024	0.006	0.221	−0.018 ~ 0.076
Low-density lipoprotein-cholesterol	0.054	0.009	0.026	< 0.001	0.037 ~ 0.071

The adjusted variables included sex, age, body mass index, smoking, diastolic blood pressure, systolic blood pressure, fasting blood glucose, log glutamate-pyruvate transaminase, log glutamate-oxaloacetate transaminase, urea nitrogen, serum creatinine, and use of lipid-lowering medications

SE, standard error; STD, standardised; CI, confidence interval

**Fig. 1:**
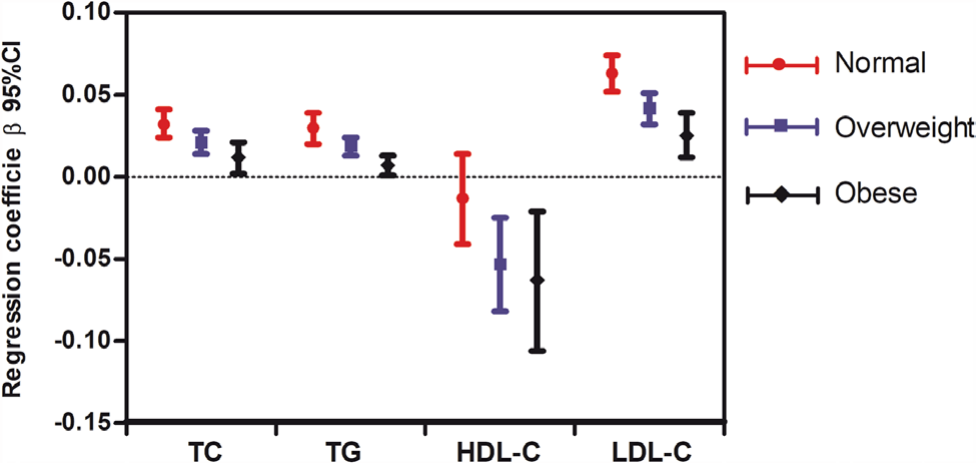
**Relationship between blood lipids and log-transformed eosinophil counts in different BMI subgroups** Adjusted variables included sex, age, body mass index, WBCcounts, smoking, diastolic blood pressure, systolic blood pressure, fasting blood glucose, log glutamate-pyruvate transaminase, log glutamate-oxaloacetate transaminase, urea nitrogen, serum creatinine, and use of lipid-lowering medications. BMI, body mass index; TG, triglyceride level; TC, total cholesterol level; LDL-C, low-density lipoprotein-cholesterol level; HDL-C, high-density lipoprotein-cholesterol level

**Fig. 2:**
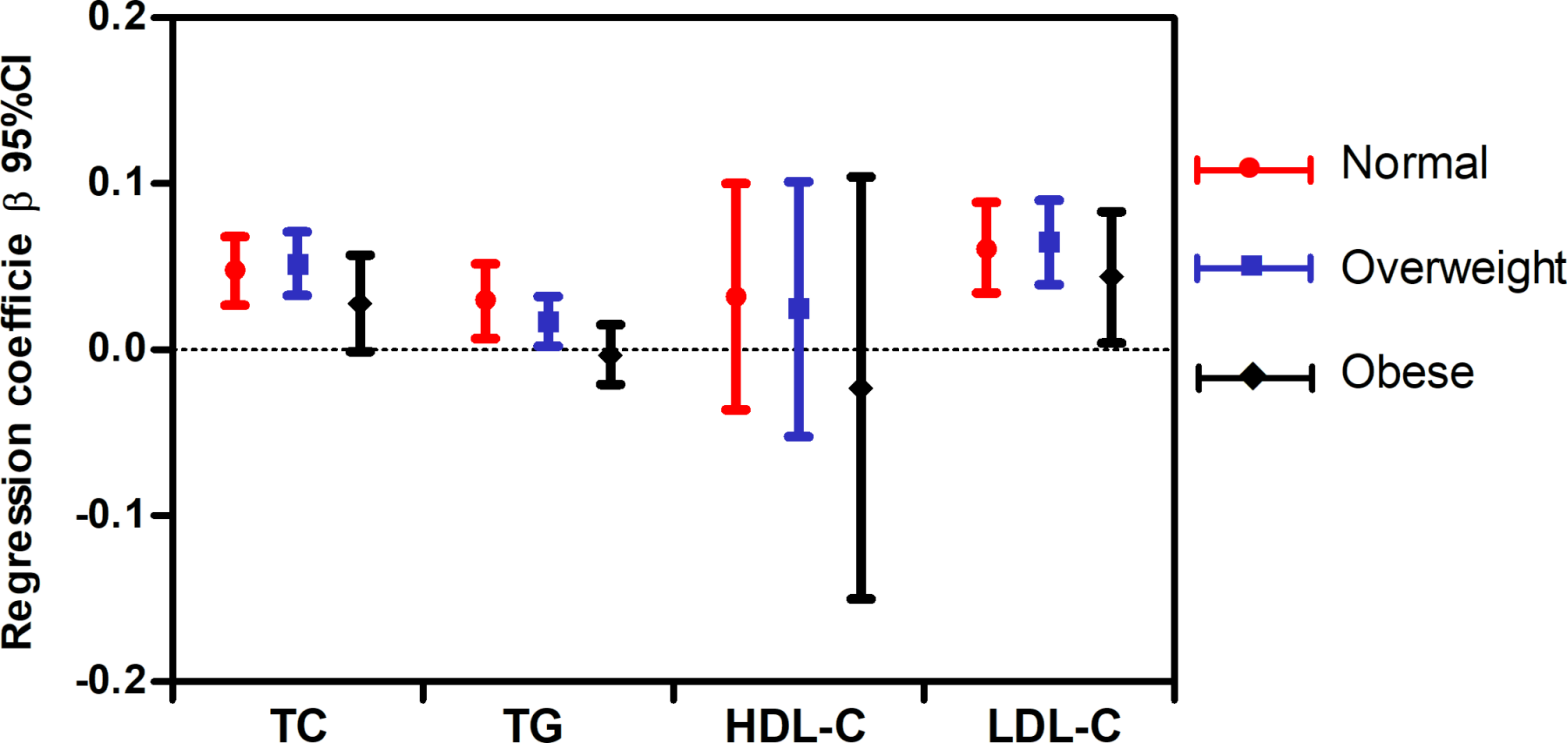
**Relationship between blood lipids and eosinophil percentage in different BMI subgroups** Adjusted variables included sex, age, body mass index, smoking, diastolic blood pressure, systolic blood pressure, fasting blood glucose, log glutamate-pyruvate transaminase, log glutamate-oxaloacetate transaminase, urea nitrogen, serum creatinine, and use of lipid-lowering medications. BMI, body mass index; TG, triglyceride level; TC, total cholesterol level; LDL-C, low-density lipoprotein-cholesterol level; HDL-C, high-density lipoprotein-cholesterol level

Figures 1 and 2 show the results of the multiple linear regression analysis for the relationship between lipid parameters and log(EOS) and eosinophil percentage, respectively, in each BMI subgroup. After adjusting for potential confounders the correlation between log(EOS) and each lipid index remained significant in most BMI status except log(EOS) was independent of HDL in the normal-weight subgroup (Fig. 1).Furthermore, the eosinophil percentage was positively correlated with TC and TG levels in the normal-weight and overweight groups but not in the obese group. Meanwhile, eosinophil percentage did not correlate with HDL-C levels in any subgroup but did with LDL-C levels in all subgroups (Fig. 2). However, there was a consistent trend in all lipid indexes, that is, with the increase of BMI, standardised β values decreased sequentially (Figs. 1 and 2).

**Fig. 3:**
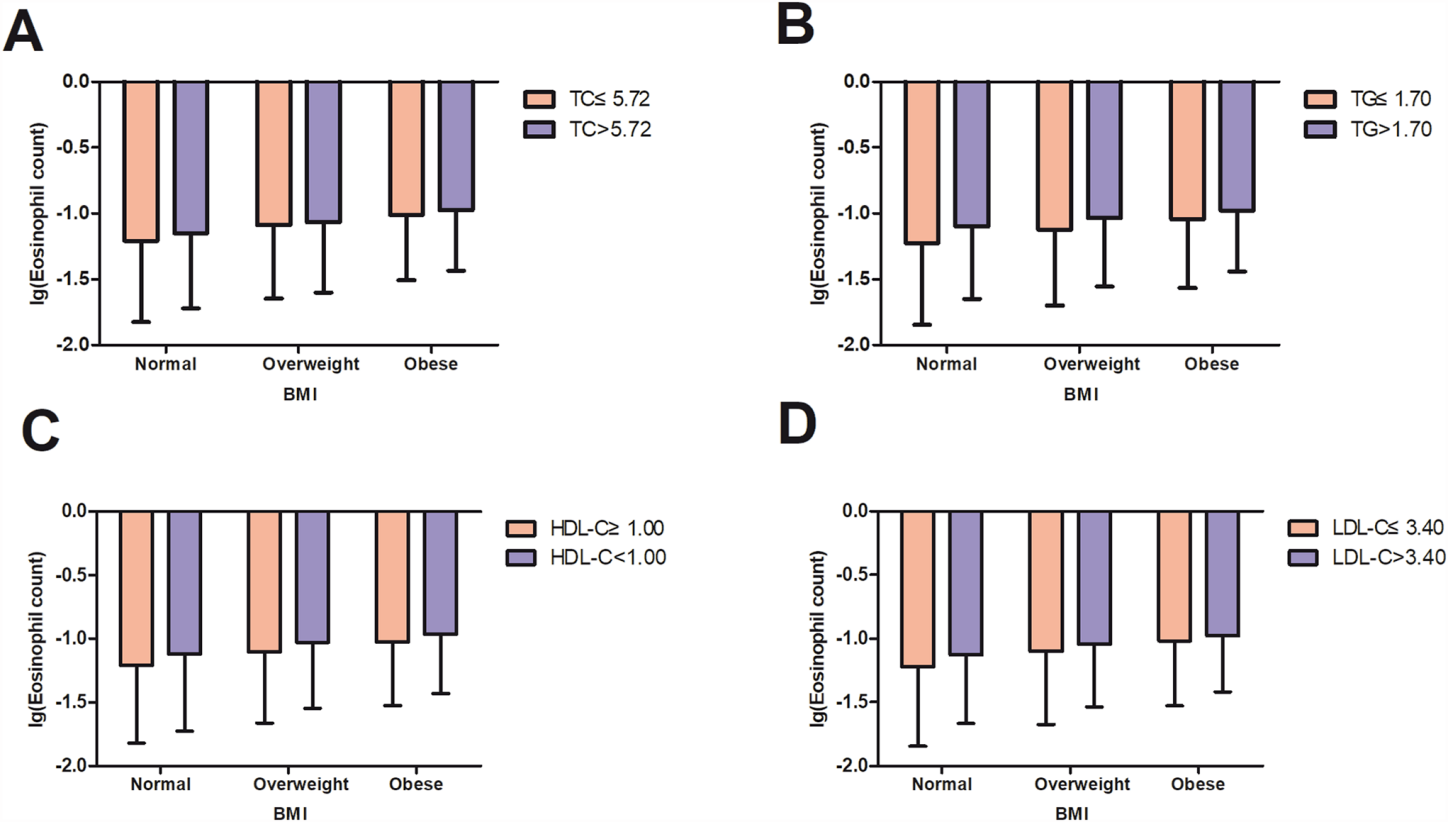
**Two-way analysis of variance regarding the effect of body mass index and serum lipids on peripheral blood eosinophil count** BMI, body mass index; TG, triglyceride level; TC, total cholesterol level; LDL-C, low-density lipoprotein-cholesterol level; HDL-C, high-density lipoprotein-cholesterol level. In A, P for TC < 0.001, P for BMI < 0.001, P for TC*BMI = 0.073;In B, P for TG < 0.001, P for BMI < 0.001, P for TG*BMI < 0.001; In C, P for HDL < 0.001, P for BMI < 0.001, P for HDL-C*BMI = 0.085; In D, P for LDL < 0.001, P for BMI < 0.001, P for LDL-C*BMI = 0.003

Figure 3 shows the ANOVA results of the effect of BMI and lipid levels on log(EOS). For each lipid variable, log(EOS) increased with higher BMI in both the normolipidaemic and hyperlipidaemic groups. Meanwhile, log(EOS) was higher in the hyperlipidaemic group than in the normolipidaemic group for all BMI subgroups (all p < 0.001). A synergistic effect exists of lipid levels (TG and LDL-C) and BMI on eosinophil count.

Considering the significant effects of gender on both eosinophil count and blood lipid, a gender -stratified analysis was performed and findings are presented in Supplementary file. In both men and women, the relationships between log(EOS) values and lipid profiles were completely consistent with that of the general population. Moreover the greater associations were observed in women because of the greater β values (Supplemental Table 1.1–1.2). Certain associations between peripheral blood eosinophil percentage and serum lipids varied by gender. Eosinophil percentage positively correlated with TC and LDL-C levels in both men and women, positively correlated with HDL-C and not with TG in men and the opposite is true in women. (Supplemental Table 2.1–2.2).

As for the BMI subgroups, regardless of log(EOS) or eosinophilic percentage, the trend of standardised β values decreased sequentially with the increase of BMI remained, especially in women (Supplemental Fig. 1.1–2.2). HDL-C had the most significant effect on eosinophils in obese women (Supplemental Fig. 1.2, 2.2). log(EOS) increased with higher BMI and hyperlipidemia whether in male or female, however a synergistic effect exists of lipid levels (TG and LDL-C) and BMI only in men, while no interaction exists in women (Supplemental Fig. 3.1–3.2).

## Discussion

There is compelling evidence that dyslipidaemia is present in respiratory inflammation and allergic diseases [6–9]. However, the few studies concerning the association between peripheral eosinophil counts and lipid levels report contradictory results. This study confirmed that in the general population, in men or in women, peripheral blood eosinophil counts are significantly positively correlated with serum LDL-C, TG, and TC levels and negatively correlated with HDL-C levels. These results are consistent with those of a prospective Lifeline’s cohort study in the Netherlands [2], a follow-up investigation of a community population in Japan [12]and studies in special population with type 2 diabetes [16] or atopic asthma [17], different from the data of a multi-ethnic cohort in the US [13] and a Caucasian population in the UK [14].This may be due to the differences in genetic background.

In addition, the survey further examined the relationship between eosinophil percentage and lipid profiles. We know, total peripheral blood leukocyte is significantly correlated with serum lipid levels [18]. Not only by adjusting total white blood cell count, we also adopt the index of eosinophil percentage, for leukocyte classification directly indicates the type of inflammation. The result found that eosinophil percentage was still correlated with LDL-C and TC levels, but the association with TG or HDL-C varied by gender. Therefore, it is necessary to consider the influence of gender when discussing the relationship between blood lipids and eosinophilic inflammation [19].

As obesity and hyperlipidaemia often coexist and because obesity is also associated with eosinophil counts [15], our study further analysed whether the relationship between lipid levels and blood eosinophils were influenced by BMI status. Similar studies are lacking in previous literature. The results from the two-factor ANOVA suggested that hyperlipidaemia and BMI levels were significantly and independently associated with higher peripheral eosinophil counts and that there was a synergistic effect between lipid levels and BMI in men. This is consistent with the finding of a study in an Austrian general population that suggested that metabolic syndrome (odds ratio, 1.41) and obesity (odds ratio, 1.16) were significantly related with elevated blood eosinophil counts and demonstrated a cumulative effect [20]. Further, our subgroup analysis of different BMI levels revealed that, lipid profiles affected blood eosinophil counts in majority subgroups and standardised β values decreased with increasing BMI. The same is true for eosinophilic percentage. This finding may be because blood neutrophil counts increase with higher BMI in an accelerated manner [15], masking the association between eosinophil and dyslipidaemia. After all, neutrophilic inflammation is dominant rather than eosinophilic inflammation in obese patients with asthma. It also may be related to the function of peripheral blood eosinophils transferring to subcutaneous tissue to prevent obesity [21]. Intriguingly, HDL is more closely associated with eosinophilic inflammation in obese individuals, especially women.This conclusion confirms the importance of HDL for immunity [22] and of great significance for clinical application. To the best of our knowledge, this is the first study to suggest that BMI status affects the correlation between lipid profiles and eosinophilic inflammation. Additionally, this result may partially explain why there was no significant association between blood lipid levels and eosinophil counts in the Multi-Ethnic Study of Atherosclerosis cohort [13] which cohort had a mean BMI of 29.3 kg/m^2^ compared to a BMI of 25.04 kg/m^2^ in the current cohort.

The Global Initiative for Asthma suggests using blood eosinophil counts to mark type 2 inflammation [23]. Our findings may help deepen the current understanding regarding the fluctuations of blood eosinophil count distribution in the general population and patients with eosinophil-related diseases. Inflammation, allergies, and lipid profiles may have the same functional network [24]. Dyslipidaemia may promote Th2 cell polarisation, establishing a chronic inflammatory state [25]. Our study supports this idea and makes several points. First, the increasing prevalence of obesity and dyslipidaemia in modern society may be one reason for the recent increase in eosinophil-related diseases, such as eosinophilic nasal polyps and allergic rhinitis. An outpatient investigation in the US also found that statin use decreased the rates of chronic rhinosinusitis [26]. Second, attention should be given to lipid metabolism, especially elevation of TG,TC,LDL-C in non-obese individuals and decline of HDL-C in obese individuals with eosinophil-related diseases. From the perspective of metabolic regulation, including controlling obesity and lowering lipid levels, the goal of controlling eosinophil-related diseases might be achieved. We intend to verify this hypothesis in future studies.

This study has some limitations and associated recommendations for further research. Firstly, it is a single-centre study, and the population was limited to Han Chinese adults. However, the large sample size presumably reduced the unbalanced distribution of confounding factors. Future multi-centre, multi-ethnic studies, and paediatric investigations are needed, as eosinophilic inflammation and dyslipidaemia are also common in children. Secondly, owing to the cross-sectional nature of this study, we could not determine the directional causation of this association. Further longitudinal studies, especially those involving the application of lipid-lowering medications, may help better understand this aspect. Finally, additional research on lipids to type 2 inflammatory cytokines, such as serum periostin or interleukin-5 levels, would help provide further valuable information regarding type 2 immunity and lipid metabolism.

## Conclusions

This study confirmed a positive association between hyperlipidaemia and peripheral blood eosinophil counts and percentages and demonstrated that the degree of this association was modified by BMI status. Furthermore, there was a synergistic effect between lipid levels and BMI on blood eosinophil counts. The findings provide new insight into the mechanisms and therapeutic approaches to eosinophil-related diseases.

### Electronic supplementary material

Below is the link to the electronic supplementary material.

Supplementary Material 1

## Data Availability

The dataset supporting the conclusions of this article is available from the corresponding author upon request.

## Abbreviations

ANOVA: Analysis of variance
BMI: Body mass index
FBG: Fasting blood glucose
HDL-C: High-density lipoprotein-cholesterol
LDL-C: Low-density lipoprotein-cholesterol
Log(EOS): Log-transformed eosinophil count
TC: Total cholesterol
TG: Triglyceride

## References

[1] Sakuma Y, Ishitoya J, Komatsu M, Shiono O, Hirama M, Yamashita Y (2011). New clinical diagnostic criteria for eosinophilic chronic rhinosinusitis. Auris Nasus Larynx.

[2] Amini M, Bashirova D, Prins BP, Corpeleijn E, LifeLines C, Study, Bruinenberg M (2016). Eosinophil count is a common factor for complex metabolic and pulmonary traits and diseases: the lifelines cohort study. PLoS ONE.

[3] Jiang WX, Cao PP, Li ZY, Zhai GT, Liao B, Lu X (2019). A retrospective study of changes of histopathology of nasal polyps in adult chinese in central China. Rhinology.

[4] Bapat SP, Whitty C, Mowery CT, Liang Y, Yoo A, Jiang Z (2022). Obesity alters pathology and treatment response in inflammatory disease. Nature.

[5] Schaftenaar F, Frodermann V, Kuiper J, Lutgens E, Atherosclerosis (2016). The interplay between lipids and immune cells. Curr Opin Lipidol.

[6] Vinding RK, Stokholm J, Chawes BLK, Bisgaard H (2016). Blood lipid levels associate with childhood asthma, airway obstruction, bronchial hyperresponsiveness, and aeroallergen sensitization. J Allergy Clin Immunol.

[7] Yang M, Yang T, Li X, Li D, Liao Z, Shen Y (2021). Clinical predictors of high blood eosinophils in chronic obstructive pulmonary disease. Int J Chron Obstruct Pulmon Dis.

[8] Sheha D, El-Korashi L, AbdAllah AM, El Begermy MM, Elzoghby DM, Elmahdi A (2021). Lipid profile and IL-17A in allergic rhinitis: correlation with disease severity and quality of life. J Asthma Allergy.

[9] Lee EJ, Kim JH, Suh YS, Choi BI, Jung CM, Kim KS (2015). Analysis of prevalence and risk factors of chronic rhinosinusitis in hyperlipidemia patients. Korean J Otorhinolaryngol-Head Neck Surg.

[10] Xie S, Jiang S, Zhang H, Wang F, Liu Y, She Y (2021). Prediction of sublingual immunotherapy efficacy in allergic rhinitis by serum metabolomics analysis. Int Immunopharmacol.

[11] Venter C, Meyer RW, Nwaru BI, Roduit C, Untersmayr E, Adel-Patient K (2019). EAACI position paper: influence of dietary fatty acids on asthma, food allergy, and atopic dermatitis. Allergy.

[12] Nishi K, Matsumoto H, Tashima N, Terada S, Nomura N, Kogo M (2021). Impacts of lipid-related metabolites, adiposity, and genetic background on blood eosinophil counts: the Nagahama study. Sci Rep.

[13] Lai YC, Woollard KJ, McClelland RL, Allison MA, Rye KA, Ong KL (2019). The association of plasma lipids with white blood cell counts: results from the multi-ethnic study of atherosclerosis. J Clin Lipidol.

[14] Tucker B, Sawant S, McDonald H, Rye KA, Patel S, Ong KL (2021). The association of serum lipid and lipoprotein levels with total and differential leukocyte counts: results of a cross-sectional and longitudinal analysis of the UK Biobank. Atherosclerosis.

[15] Sunadome H, Matsumoto H, Izuhara Y, Nagasaki T, Kanemitsu Y, Ishiyama Y (2020). Correlation between eosinophil count, its genetic background and body mass index: the Nagahama study. Allergol Int.

[16] Shim WS, Kim HJ, Kang ES, Ahn CW, Lim SK, Lee HC (2006). The association of total and differential white blood cell count with metabolic syndrome in type 2 diabetic patients. Diabetes Res Clin Pract.

[17] Barochia AV, Gordon EM, Kaler M, Cuento RA, Theard P, Figueroa DM (2017). High density lipoproteins and type 2 inflammatory biomarkers are negatively correlated in atopic asthmatics. J Lipid Res.

[18] Liu Y, Kong X, Wang W, Fan F, Zhang Y, Zhao M (2017). Association of peripheral differential leukocyte counts with dyslipidemia risk in chinese patients with hypertension: insight from the China Stroke Primary Prevention Trial. J Lipid Res.

[19] Catherine J, Andersen,Terrence M, Vance (2019). Gender dictates the relationship between serum lipids and leukocyte counts in the National Health and Nutrition Examination Survey 1999-2004. J Clin Med.

[20] Hartl S, Breyer MK, Burghuber OC, Ofenheimer A, Schrott A, Urban MH (2020). Blood eosinophil count in the general population: typical values and potential confounders. Eur Respir J.

[21] Maizels RM, Allen JE (2011). Immunology. Eosinophils forestall obesity. Science.

[22] Marsche AT et al. High-Density Lipoprotein (HDL) in Allergy and Skin Diseases: Focus on Immunomodulating Functions. Biomedicines. 2020;8:558 – 83 10.3390/biomedicines8120558. 10.3390/biomedicines8120558 PMC7760586 33271807

[23] GINA. Global Initiative for Asthma. Global Strategy for Asthma Management and Prevention. www.ginasthma.org. Accessed 10 May 2019.

[24] La Mantia I, Andaloro C, Albanese PG, Varricchio A (2017). Blood lipid levels related to allergic rhinitis: a significant association. Euro Mediterr Biomed J.

[25] Hu X, Wang Y, Hao LY, Liu X, Lesch CA, Sanchez BM (2015). Sterol metabolism controls T(H)17 differentiation by generating endogenous RORγ agonists. Nat Chem Biol.

[26] Wilson JH, Payne SC, Fermin CR, Churnin I, Qazi J, Mattos JL (2020). Statin use protective for chronic rhinosinusitis in a nationally representative sample of the United States. Laryngoscope.

