# Supplementary material for: Body mass index affects the association between plasma lipids and peripheral eosinophils in a general chinese population: a cross-sectional survey
Source: Lipids Health Dis. 2023 Sep 7;22:146. doi: 10.1186/s12944-023-01909-w (PMC10483721; doi:10.1186/s12944-023-01909-w)
Supplement: Supplementary file 1 — Supplementary Material 1 [file 12944_2023_1909_MOESM1_ESM.docx]

**Supplemental Table 1.1 Multiple linear regression results of log-transformed eosinophil count with lipid parameters in men**

| Independent variable | β | SE | STDβ | P | 95% CI |
| --- | --- | --- | --- | --- | --- |
| Total cholesterol | 0.019 | 0.003 | 0.032 | <0.001 | 0.012~0.025 |
| Triglycerides | 0.011 | 0.002 | 0.029 | <0.001 | 0.007~0.016 |
| High-density lipoprotein-cholesterol | −0.040 | 0.013 | −0.018 | 0.002 | -0.065~ -0.015 |
| Low-density lipoprotein-cholesterol | 0.038 | 0.004 | 0.048 | <0.001 | 0.029~0.046 |

The adjusted variables included age, body mass index, WBC count, smoking, diastolic blood pressure, systolic blood pressure, fasting blood glucose, log glutamate-pyruvate transaminase, log glutamate-oxaloacetate transaminase, urea nitrogen, serum creatinine, and use of lipid-lowering medications.

SE, standard error; STD, standardised; CI, confidence interval

**Supplemental Table 1.2 Multiple linear regression results of log-transformed eosinophil count with lipid parameters in women**

| Independent variable | β | SE | STDβ | P | 95% CI |
| --- | --- | --- | --- | --- | --- |
| Total cholesterol | 0.026 | 0.004 | 0.043 | <0.001 | 0.019~0.034 |
| Triglycerides | 0.033 | 0.004 | 0.050 | <0.001 | 0.025~0.042 |
| High-density lipoprotein-cholesterol | −0.033 | 0.013 | −0.016 | 0.013 | -0.060~ -0.007 |
| Low-density lipoprotein-cholesterol | 0.055 | 0.005 | 0.068 | <0.001 | 0.045~0.065 |

The adjusted variables included age, body mass index, WBCcount, menopausal status, smoking, diastolic blood pressure, systolic blood pressure, fasting blood glucose, log glutamate-pyruvate transaminase, log glutamate-oxaloacetate transaminase, urea nitrogen, serum creatinine, and use of lipid-lowering medications.

SE, standard error; STD, standardised; CI, confidence interval

**Supplemental Table 2.1 Multiple linear regression results of eosinophil percentage with lipids**

**parameters in men.**

| Independent variable | β | SE | STDβ | P | 95% CI |
| --- | --- | --- | --- | --- | --- |
| Total cholesterol | 0.050 | 0.009 | 0.030 | <0.001 | 0.032~0.068 |
| Triglycerides | 0.008 | 0.006 | 0.007 | 0.194 | -0.004~0.021 |
| High-density lipoprotein-cholesterol | 0.088 | 0.037 | 0.013 | 0.018 | 0.015~0.161 |
| Low-density lipoprotein-cholesterol | 0.062 | 0.012 | 0.027 | <0.001 | 0.037 ~ 0.086 |

The adjusted variables included age, body mass index, smoking, diastolic blood pressure, systolic blood pressure, fasting blood glucose, log glutamate-pyruvate transaminase, log glutamate-oxaloacetate transaminase, urea nitrogen, serum creatinine, and use of lipid-lowering medications.

SE, standard error; STD, standardised; CI, confidence interval.

**Supplemental Table 2.2 Multiple linear regression results of eosinophil percentage with lipids**

**parameters in women.**

| Independent variable | β | SE | STDβ | P | 95% CI |
| --- | --- | --- | --- | --- | --- |
| Total cholesterol | 0.040 | 0.009 | 0.030 | <0.001 | 0.022~0.057 |
| Triglycerides | 0.029 | 0.010 | 0.020 | 0.004 | 0.009~0.048 |
| High-density lipoprotein-cholesterol | -0.023 | 0.030 | -0.005 | 0.442 | −0.081~0.035 |
| Low-density lipoprotein-cholesterol | 0.053 | 0.012 | 0.030 | <0.001 | 0.030 ~ 0.076 |

The adjusted variables included age, body mass index, menopausal status, smoking, diastolic blood pressure, systolic blood pressure, fasting blood glucose, log glutamate-pyruvate transaminase, log glutamate-oxaloacetate transaminase, urea nitrogen, serum creatinine, and use of lipid-lowering medications.

SE, standard error; STD, standardised; CI, confidence interval.

**Supplemental Fig 1.1. Relationship between blood lipids and log-transformed eosinophil counts in different body mass index subgroups in men.**

**
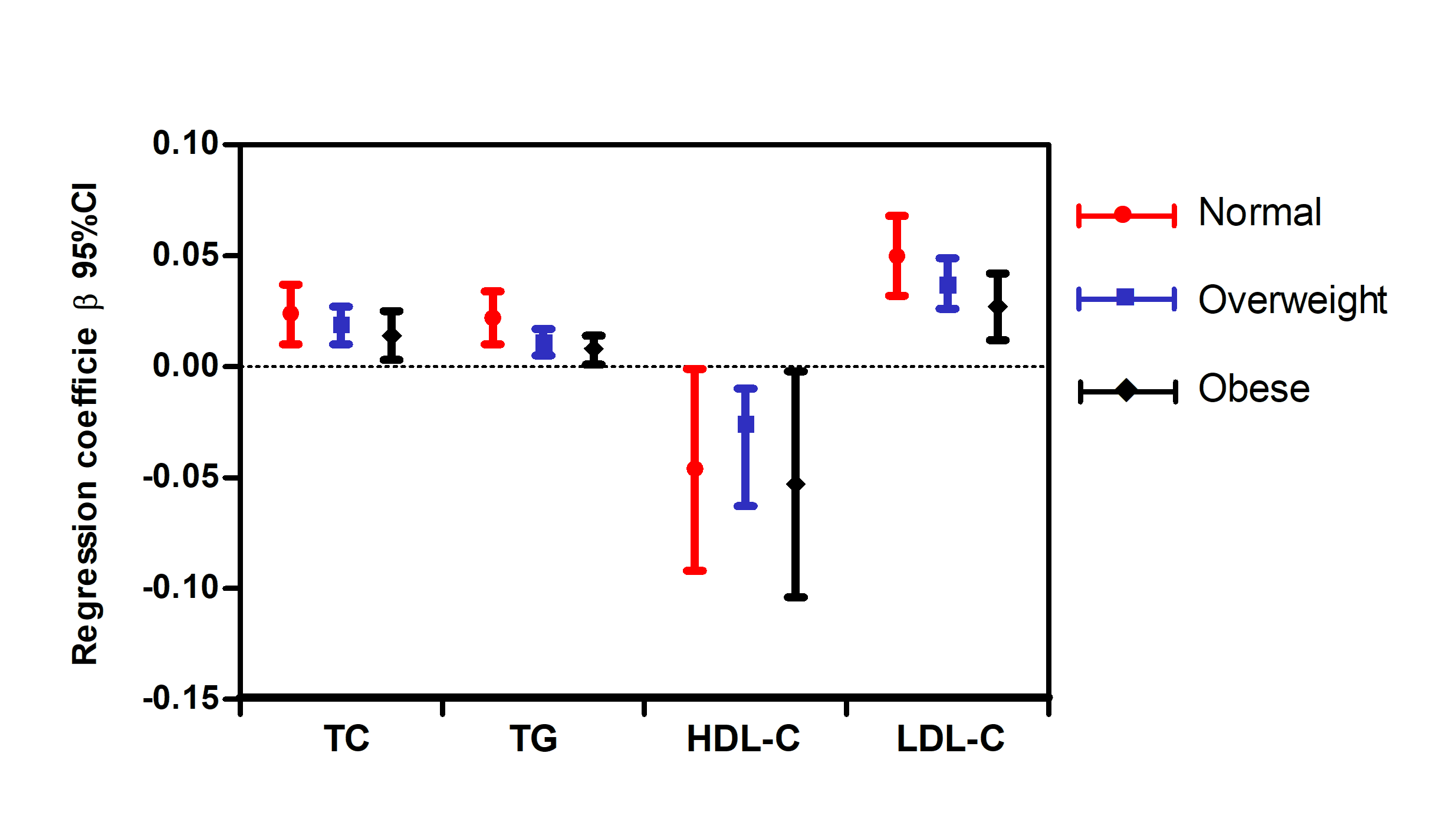
**

Adjusted variables included age, body mass index, WBC counts, smoking, diastolic blood pressure, systolic blood pressure, fasting blood glucose, log glutamate-pyruvate transaminase, log glutamate-oxaloacetate transaminase, urea nitrogen, serum creatinine, and use of lipid-lowering medications. BMI, body mass index; TG, triglyceride level; TC, total cholesterol level; LDL-C, low-density lipoprotein-cholesterol level; HDL-C, high-density lipoprotein-cholesterol level.

**Supplemental Fig 1.2. Relationship between blood lipids and log-transformed eosinophil counts in different body mass index subgroups in women.**

**
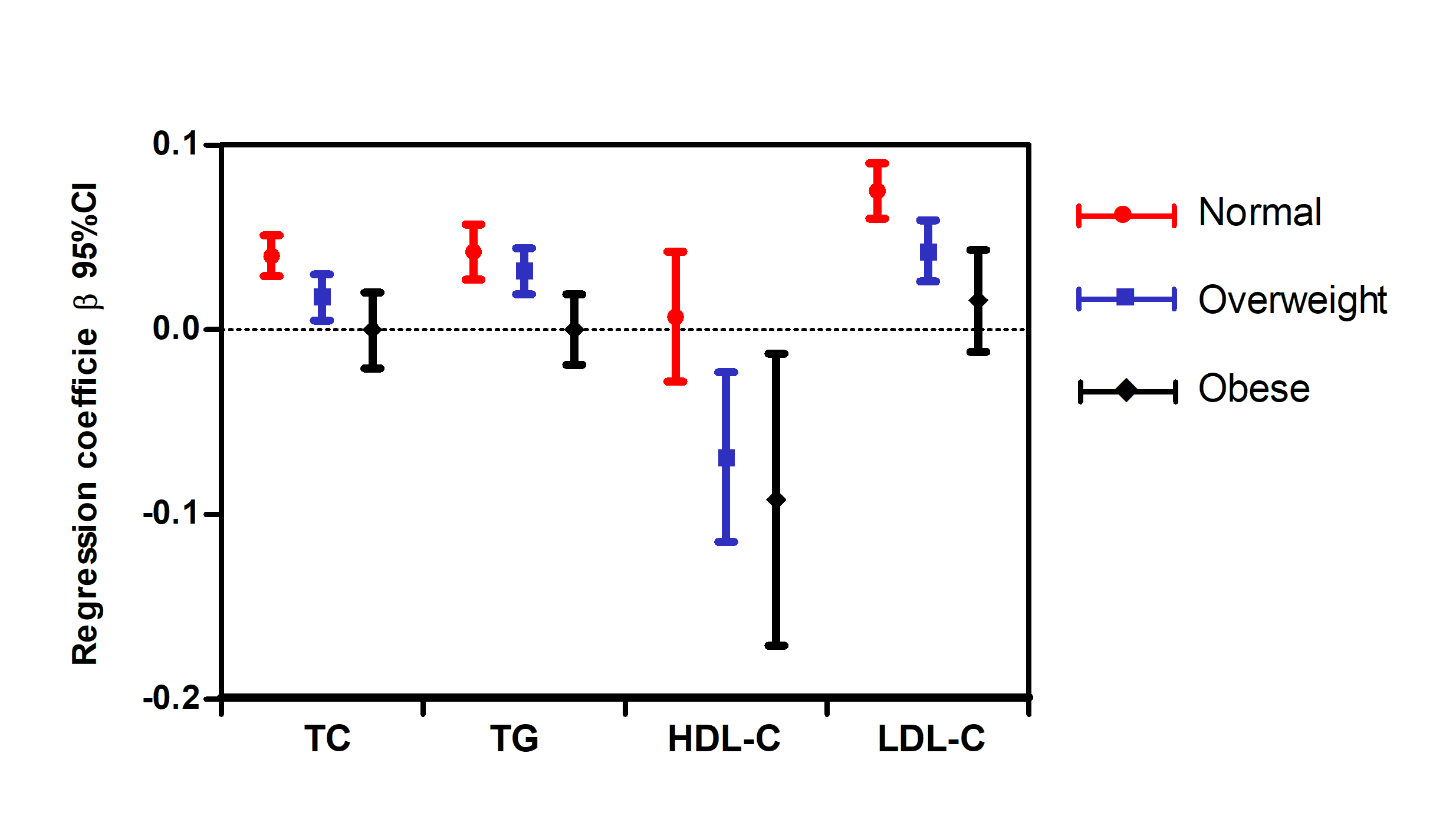
**

Adjusted variables included age, body mass index, WBC counts, menopausal status, smoking, diastolic blood pressure, systolic blood pressure, fasting blood glucose, log glutamate-pyruvate transaminase, log glutamate-oxaloacetate transaminase, urea nitrogen, serum creatinine, and use of lipid-lowering medications. BMI, body mass index; TG, triglyceride level; TC, total cholesterol level; LDL-C, low-density lipoprotein-cholesterol level; HDL-C, high-density lipoprotein-cholesterol level.

**Supplemental Fig 2.1. Relationship between lipids and eosinophil percentage in different BMI subgroups in men.**

**
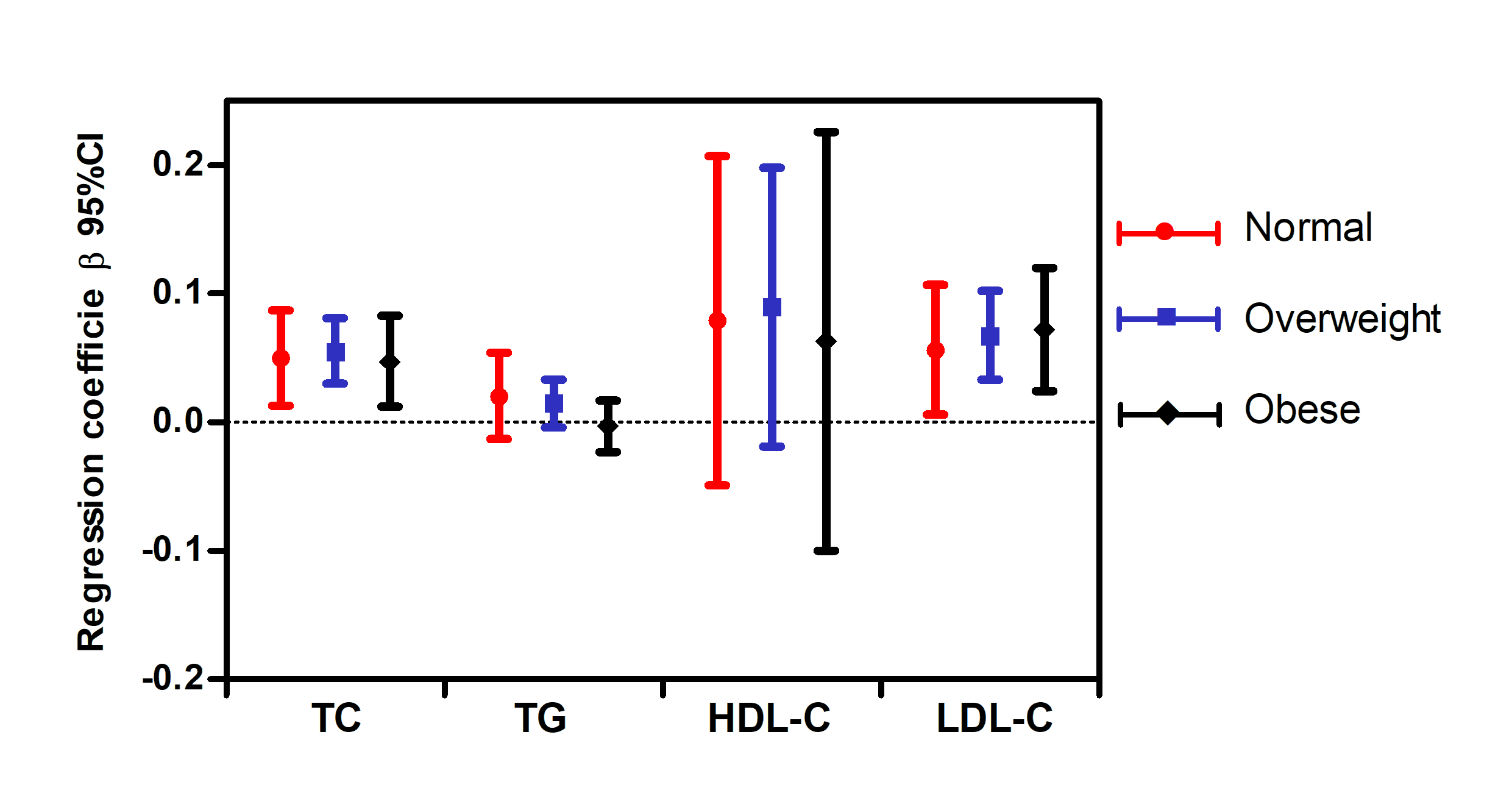
**

Adjusted variables included age, body mass index, smoking, diastolic blood pressure, systolic blood pressure, fasting blood glucose, log glutamate-pyruvate transaminase, log glutamate-oxaloacetate transaminase, urea nitrogen, serum creatinine, and use of lipid-lowering medications. BMI, body mass index; TG, triglyceride level; TC, total cholesterol level; LDL-C, low-density lipoprotein-cholesterol level; HDL-C, high-density lipoprotein-cholesterol level.

**Supplemental Fig 2.2. Relationship between lipids and eosinophil percentage in different BMI subgroups in women.**

**
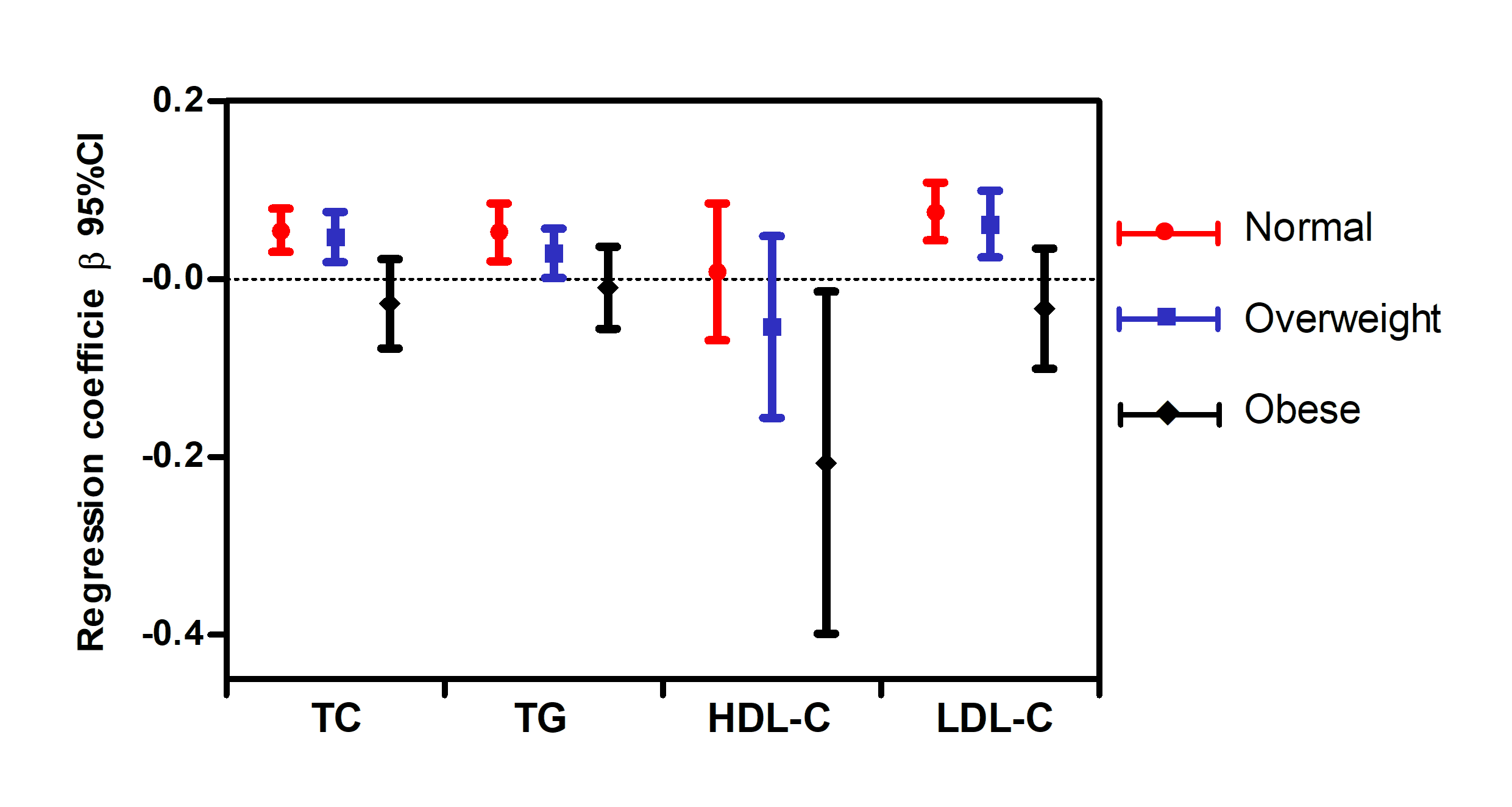
**

Adjusted variables included age, body mass index, menopausal status, smoking, diastolic blood pressure, systolic blood pressure, fasting blood glucose, log glutamate-pyruvate transaminase, log glutamate-oxaloacetate transaminase, urea nitrogen, serum creatinine, and use of lipid-lowering medications. BMI, body mass index; TG, triglyceride level; TC, total cholesterol level; LDL-C, low-density lipoprotein-cholesterol level; HDL-C, high-density lipoprotein-cholesterol level.

**Supplemental Fig 3.1. Two-way analysis of variance regarding the effect of body mass index and serum lipids on peripheral blood eosinophil count in men.**

**
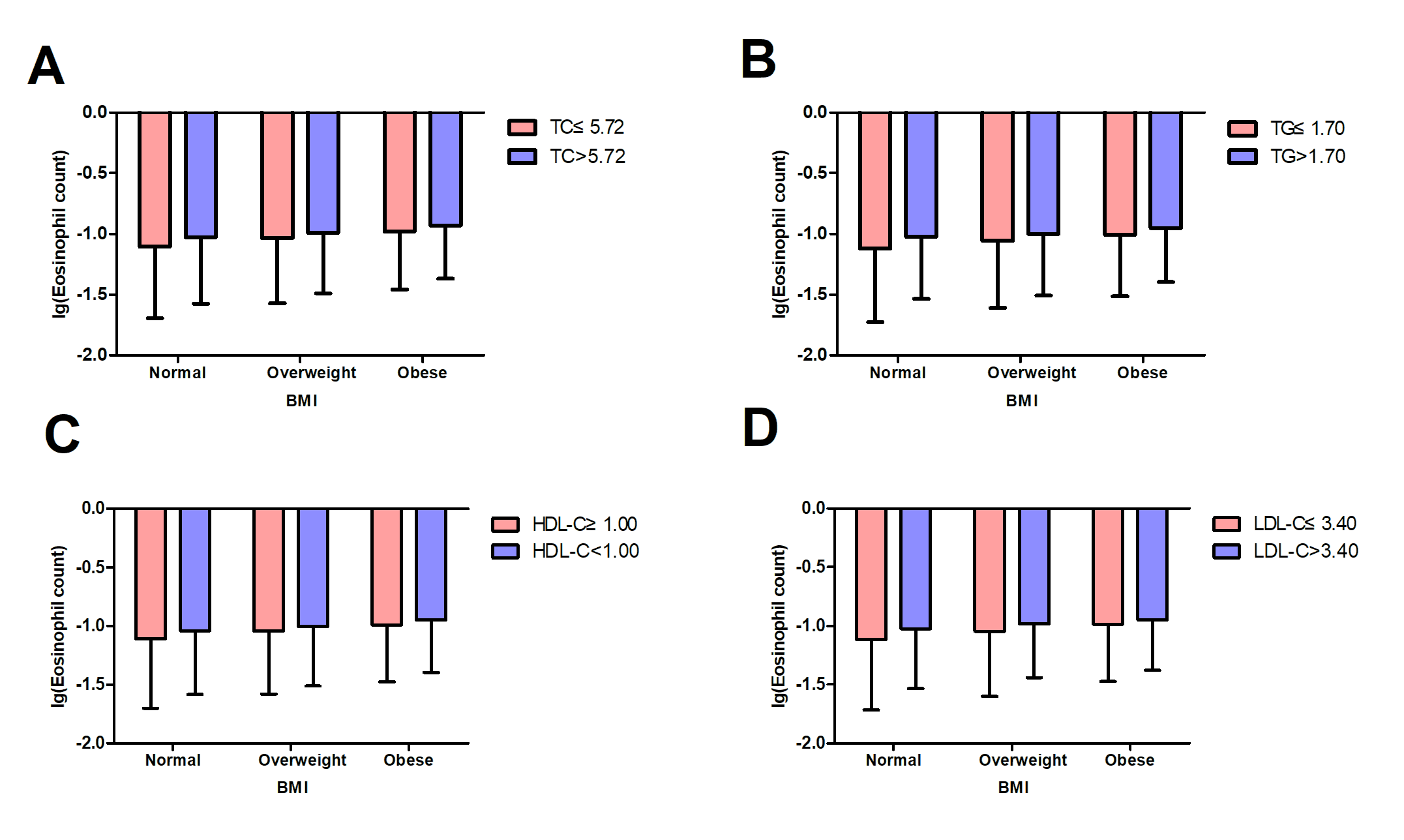
**

BMI, body mass index; TG, triglyceride level; TC, total cholesterol level; LDL-C, low-density lipoprotein-cholesterol level; HDL-C, high-density lipoprotein-cholesterol level.

In A，P for TC <0.001，P for BMI <0.001，P for TC*BMI=0.349；

In B，P for TG <0.001，P for BMI <0.001，P for TG*BMI =0.014；

In C，P for HDL <0.001，P for BMI <0.001，P for HDL-C*BMI=0.228；

In D，P for LDL <0.001，P for BMI <0.001，P for LDL-C*BMI=0.022

**Supplemental Fig 3.2. Two-way analysis of variance regarding the effect of body mass index and serum lipids on peripheral blood eosinophil count in women.**

**
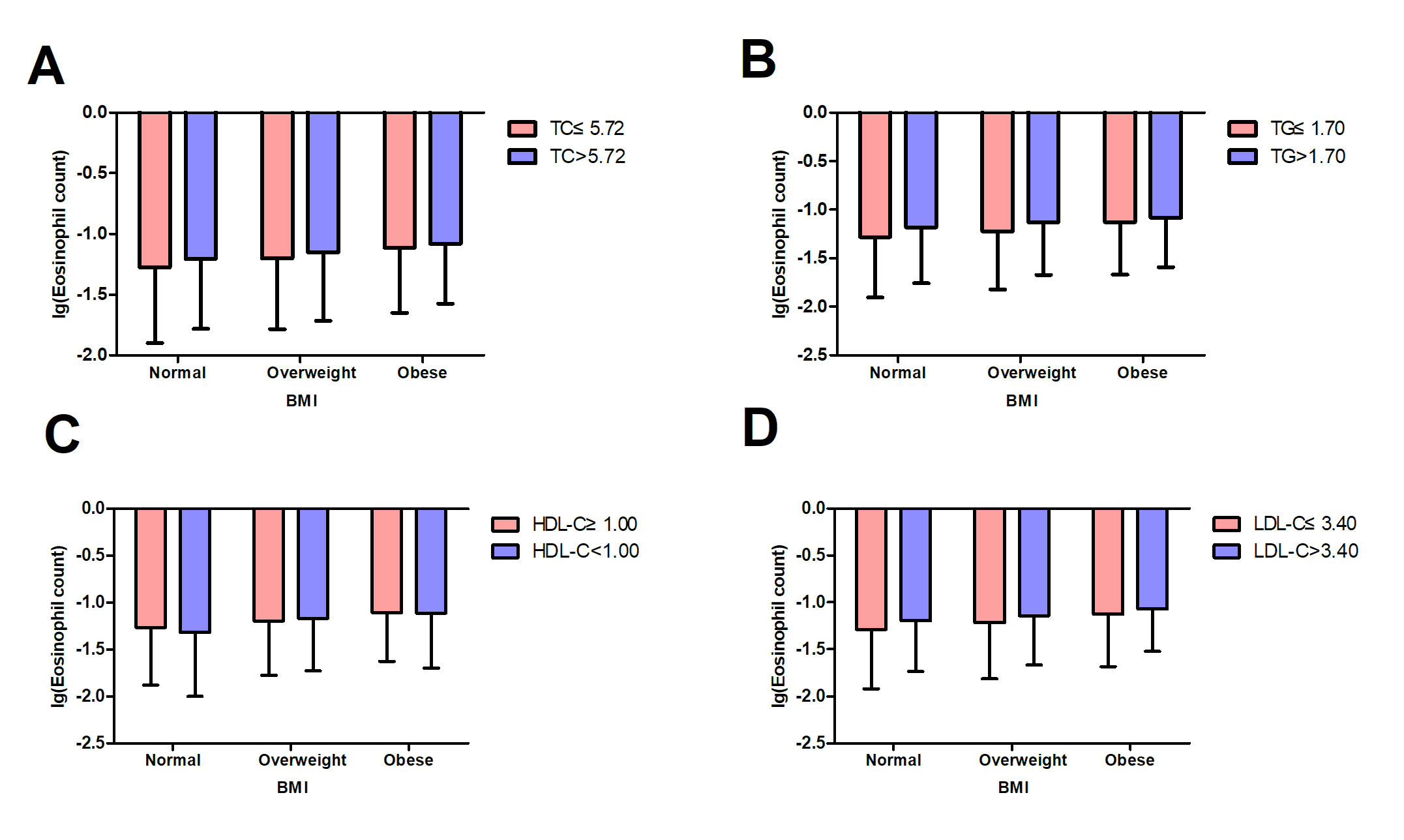
**

BMI, body mass index; TG, triglyceride level; TC, total cholesterol level; LDL-C, low-density lipoprotein-cholesterol level; HDL-C, high-density lipoprotein-cholesterol level.

In A，P for TC<0.001，P for BMI<0.001，P for TC*BMI=0.416；

In B，P for TG <0.001，P for BMI <0.001，P for TG*BMI =0.108；

In C，P for HDL =0.532，P for BMI <0.001，P for HDL-C*BMI=0.077；

In D，P for LDL <0.001，P for BMI <0.001，P for LDL-C*BMI=0.252
